# A rare and fatal case of tubo-ovarian abscess due to *Burkholderia pseudomallei* presenting as puerperal sepsis

**DOI:** 10.1099/acmi.0.000500.v3

**Published:** 2023-09-07

**Authors:** Maanasa Bhaskar M, Soundarya Rajamanikam, Sruthi Raj, Sujatha Sistla, Kubera Siddappa Nichanahalli

**Affiliations:** ^1^​ Department of Microbiology, Jawaharlal Institute of Post Graduate Medical Education and Research, Puducherry 605006, India; ^2^​ Department of Obstetrics and Gynaecology, Jawaharlal Institute of Post Graduate Medical Education and Research, Puducherry 605006, India

**Keywords:** melioidosis, puerperal sepsis, tubo-ovarianabscess

## Abstract

**Introduction.:**

Melioidosis is an emerging life-threatening infectious disease caused by the Gram-negative *Burkholderia pseudomallei* found in contaminated soil and surface ground water. It often presents with varied clinical manifestations and has a high mortality if left untreated due to lack of clinical suspicion. Here we report a rare and fatal case of tubo-ovarian abscess presenting as puerperal sepsis due to *B. pseudomallei* in a young woman.

**Case report.:**

A 25-year-old female presented for medical consultation at day 43 postpartum complaining of fever on and off for the past 40 days associated with vomiting. On per speculum examination, greenish discharge from the cervix was seen and a right adnexal mass was felt which was cystic in consistency, non-tender and pushing the cervix to the left side. An exploratory laparotomy was carried out and pus was drained from below the rectus sheath, and from the right tubo-ovarian mass. Peripheral blood and the pus samples collected intraoperatively grew *B. pseudomallei *. The patient died on the fifth post-operative day due to septic shock with disseminated intravascular coagulation secondary to puerperal sepsis.

**Conclusion.:**

Melioidosis is a fatal but treatable disease when it is promptly diagnosed. In countries such as India, where tuberculosis is highly endemic, underdiagnosis of melioidosis can be common. Clinicians and microbiologists should have a high index of suspicion of melioidosis especially in individuals with underlying illness.

## Data Summary

No data was generated during this research or is required for the work to be reproduced.

## Introduction

Melioidosis is an emerging life-threatening infection caused by the Gram-negative bacterium *Burkholderia pseudomallei* which is found in the rhizosphere and surface ground water. Melioidosis, popularly known as the ‘Great Mimicker’, manifests clinically in different forms with varying clinical severity including chronic localized abscess mimicking tuberculosis, osteomyelitis, pneumonia, sepsis and even seroconversion without any clinically evident illness [1–7]. The global distribution of melioidosis was initially confined to some parts of Southeast Asia and Northern Australia (i.e. tropical regions falling between 20°N and 20°S) including Thailand, Malaysia, Cambodia and Vietnam, but is now spreading slowly to other tropical regions of the world such as China, Sri Lanka, Brazil and India [8, 9]. Puerperal sepsis due to *B. pseudomallei* is a rare clinical entity even in countries where *B. pseudomallei* is endemic. There are no case reports of puerperal sepsis due to *B. pseudomallei* in the Indian population and hence we report the first case.

## Case presentation

A 25-year-old female, with one live birth and one previous miscarriage, who had undergone an emergency caesarean section at 35 weeks of gestation in view of severe oligohydramnios and intra-uterine fetal growth retardation, presented to the emergency department on day 43 postpartum with fever on and off since day 1 post-surgery. Fever was high grade and intermittent in nature. There was no history of burning micturition, night sweats or evening rise of temperature. Her antenatal period had been uneventful.

The patient was initially treated at a private hospital for fever where she was given ceftazidime 2 g 8-hourly for 7 days. Her symptoms improved and she was discharged. However, in view of a recurrence of fever associated with vomiting and worsening of her general condition, she was referred to our tertiary care centre for further evaluation.

On examination, the patient was well built and moderately nourished, conscious, obeying commands but appeared disoriented with irrelevant talk. Pallor was present with mild bilateral pitting pedal oedema. There was no icterus, cyanosis or lymphadenopathy. Physical examination revealed temperature of 101.2 °F (38.4 °C), pulse rate of 138 beats min^–1^, blood pressure of 108/66 mmHg and respiratory rate of 30 min^–1^ with oxygen saturation of 96 % in room air. On abdominal examination, a lower segment caesarean section (LSCS) scar was present and was found to be healthy. Mild tenderness was present in the right hypochondrium with localized guarding. The uterus was not palpable and no organomegaly was detected. Bowel sounds were present. Other systemic examination results were found to be unremarkable.

On per speculum examination, greenish discharge from the cervix was seen. On bimanual examination, the uterus was found to be of 8–10 weeks in size, mobile and anteverted. A right adnexal mass measuring 6×7 cm was felt which was cystic in consistency, non-tender and pushing the cervix to the left side.

## Investigations

**Fig. 1.**
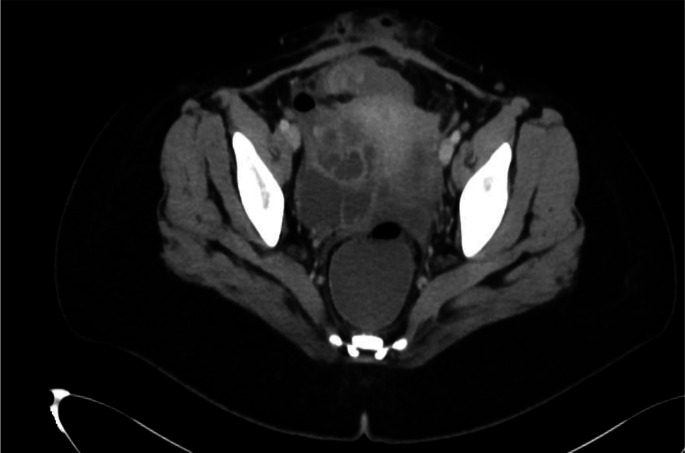
CECT abdomen and pelvis showing a right side tubo-ovarian abscess.

Laboratory investigations revealed low haemoglobin (9.2 g l^–1^), total leucocyte count of 17×10^3^ cells µl^–1^ with neutrophilic predominance and a platelet count of 2×10^5^ cells µl^–1^ . Contrast enhanced computer tomography (CECT) of the abdomen and pelvis showed the presence of a multiseptated cystic lesion measuring 5×4 cm in the right adnexa with no solid component (Fig. 1). The uterus was of normal size with no intra-uterine collection. A provisional diagnosis of right tubo-ovarian abscess was made, and the patient was taken for emergency exploratory laparotomy.

Intraoperative findings were as follows. On opening the abdomen, around 5 ml of pus collection was noted below the rectus sheath. A tubo-ovarian mass measuring approximately 6×8 cm was present in the right side and around 30 ml of frank pus was drained. A right salphingo-oophorectomy was done. In total, 25–30 ml of pus was noted in the uterovaginal fold of the peritoneum. Her bladder was found to be densely adherent to the anterior wall of the uterus which was friable to touch. The omentum was found to be adherent to the posterolateral wall of the uterus. The appendix was adherent to the right cornua. Sharp dissection of the adhesions was done and a small perforation of the appendix measuring 0.5×0.5 cm was noted. Feculent material was drained from the appendix. After releasing the appendicular adhesion from the tubo-ovarian mass, an appendicectomy was performed.

**Fig. 2.**
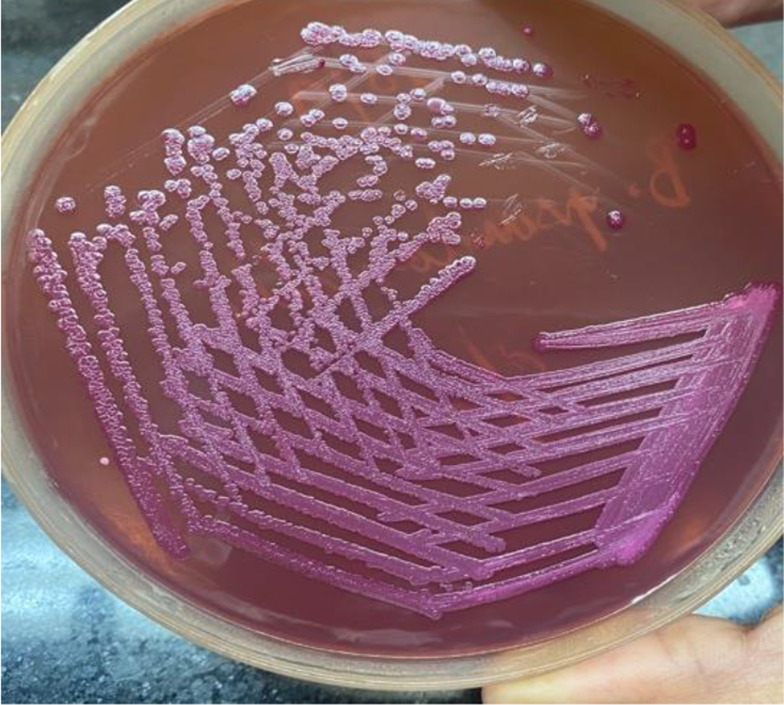
Mac Conkey agar showing non-lactose-fermenting colonies with a metallic sheen.

**Fig. 3.**
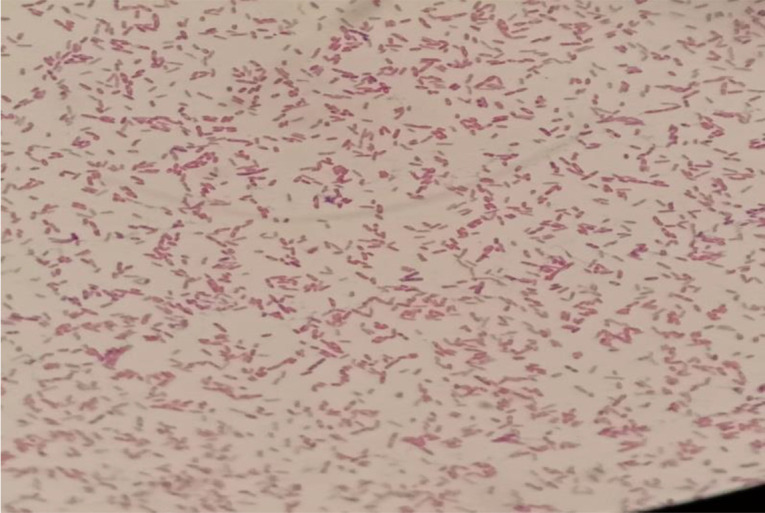
Gram-negative bacilli with bipolar staining.

Pus samples collected intraoperatively were sent for bacterial and fungal culture and Cartridge-based nucleic acid amplification (CBNAAT) for *Mycobacterium tuberculosis* complex. Simultaneously, a peripheral blood sample and high vaginal swab were also sent for culture and sensitivity testing. Blood culture grew *B. pseudomallei* which was sensitive to ceftazidime, meropenem and co-trimoxazole. Gram staining of pus samples sent for culture showed many pus cells and Gram-negative bacilli. The sample was inoculated onto 5% sheep blood agar and MacConkey agar and incubated aerobically at 37°C. Culture plates were examined after 24 h of aerobic incubation for any growth. On blood agar, a single type of colony was seen which was around 1–2 mm in size, moist and non-haemolytic, with entire margins. On MacConkey agar, non-lactose-fermenting colonies with a metallic sheen were seen (Fig. 2). Gram staining from the colonies revealed Gram-negative bacilli with bipolar staining (Fig. 3) which were catalase-positive and oxidase-positive. Colonies were subjected to identification by Vitek 2 (bioMérieux, France) and identified as *B. pseudomallei* which was found to be sensitive to ceftazidime, meropenem and co-trimoxazole. The high vaginal swab also grew *B. pseudomallei* with similar sensitivity pattern. CBNAAT for *M. tuberculosis* complex was negative.

## Treatment

Post-operatively, intravenous cefoperazone sulbactam 2 g 12-hourly, amikacin 375 mg 12-hourly and metronidazole 500 mg 12-hourly was started. During the immediate post-operative period, the patient was afebrile and conscious but continued to be tachypnoeic with a respiratory rate of 56 min^−1^ with oxygen saturation of 95 % with 6 litres of oxygen via a facemask and tachycardia of 150 beats min^–1^. She was started on intravenous fluids in view of decreased urine output. On post-operative day 2, her laboratory parameters showed thrombocytopenia, deranged coagulation profile and renal parameters, and 4 units of fresh frozen plasma was transfused in view of suspected disseminated intravascular coagulation (DIC). The patient was started on intravenous meropenem 1 g 8-hourly based on the sensitivity report. However, the patient’s condition did not improve and she had persistant tachypnoea with laboured breathing and tachycardia. She was intubated and put on ventilatory support. Blood gas analysis revealed a compensated metabolic acidosis. The patient died on the fifth post-operative day due to septic shock with disseminated intravascular coagulation secondary to puerperal sepsis.

## Discussion

*B. pseudomallei* is a Gram-negative, intracellular, aerobic, non-spore-forming bacilli and is transmitted to humans by inhalation or inoculation through skin abrasion on contact with contaminated soil or water. This organism has been isolated from endemic areas of Thailand and Northern Australia but is now spreading slowly to other tropical regions of the world such as China, Sri Lanka, Brazil and India. WPRO IRIS estimates a global burden of 165 000 human melioidosis cases with 89 000 deaths per year. They also estimate that melioidosis is underreported in about 45 endemic countries and it could be endemic in another 34 countries from where it is not yet been reported [10]. Melioidosis is not a notifiable disease in India. Hence the true incidence of the disease is unknown, and it may vary from state to state with a few hotspots within the same state. For the past few decades, India has faced increased case reports of melioidosis especially in the southern coastal regions including Malabar region of Kerala, Karnataka, Maharashtra, Tamil Nadu and Pondicherry [1, 4, 6, 11–15]. There are quite a few studies on the isolation of *B. pseudomallei* from soil from coastal regions of South India including Karnataka and Tamil Nadu, indicating that this organism could be endemic there although few cases have been reported. This could be due to underdiagnosis of melioidosis in India because of decreased awareness among clinicians, unavailability of appropriate diagnostic methods, low index of suspicion due to its varied presentation and inadequate research about this organism. Recently, Prakash *et al*. revealed the presence of *B. pseudomallei* in soil samples from paddy fields near Cuddalore district, south India. In our case, the patient was from Cuddalore district of Tamil Nadu, which is an endemic area for this organism [14]. There is a positive association between disease incidence and environmental contamination with *B. pseudomallei *.

Melioidosis is typically a community-acquired infectious disease. Though melioidosis can affect healthy individuals, severe infection is more common in individuals with predisposing factors. Diabetes mellitus, chronic alcoholism, cirrhosis, immunosuppression, chronic lung disease, chronic kidney disease, malnutrition and thalassaemia are some of the risk factors. Occupational or recreational exposure to contaminated soil or water is the usual infecting event through penetrating wounds or pre-existing wounds. However, an absence of risk factors should not exclude the possibility of melioidosis especially if the patient is from an endemic area. In our case, though pregnancy is not a recognized risk factor for melioidosis, this along with endemic geographical location would have added to the risk of acquiring the infection in the absence of other risk factors. There is very little information on the association of pregnancy with melioidosis and outcomes associated with it. It is unknown whether pregnant women are more susceptible to infection, or whether they experience more severe disease compared to non-pregnant women. Reported cases in pregnancy include a case of perinatal transmission at 32 weeks of gestation where melioidosis was diagnosed postpartum, a fatal case of bronchopneumonia complicated with spontaneous abortion at 16 weeks of gestation, a case of chorioamnionitis with preterm delivery at 23 weeks of gestation and a case of bacteraemic melioidosis with splenic abscess in a young healthy woman complicated with early fetal loss at 6 weeks of gestation [16–19].

The clinical spectrum of melioidosis is complex and wide-ranging, making it challenging to diagnose and treat [20]. The clinical manifestation ranges from septicaemia with high mortality to chronic debilitating abscesses. In our case, the patient had a tubo-ovarian abscess which probably ruptured resulting in seeding of the organism into the peritoneal cavity and subsequent bacteraemic melioidosis and sepsis leading to multi-organ dysfunction syndrome (MODS). This can be considered as a case of puerperal sepsis due to its occurrence within 42 days following delivery. A literature search did not reveal any cases of puerperal sepsis due to *B. pseudomallei* globally.

There can be a prolonged period of latency between exposure to the pathogen and disease manifestation with melioidosis. Here, the patient had severe oligohydramnios and intrauterine growth restriction which could have been due to placental infection due to *B. pseudomallei *. A live male baby weighing 1.4 kg was delivered at 35 weeks of gestation and had a clinical course in a neonatal intesive care unit which was consistent with prematurity and suspected sepsis and then discharged 30 days after birth. Since antenatal and neonatal records were not available, we could not find further details regarding this. A systematic review on neonatal outcomes in melioidosis identified pneumonia, bacteraemia and meningitis to be the usual manifestations in neonates [21]. The various possible routes of infections can be transplacental, through breast milk, community-acquired post-partum and healthcare-associated exposures.

Though this patient was initially empirically treated with ceftazidime, it was given only for a period of 7 days leading to incomplete clearance of the organism. The treatment regimen for melioidosis includes initial intravenous antimicrobial therapy with meropenem or ceftazidime for a period of 14 days followed by a continuation phase of 3–6 months of oral antibiotics consisting of co-trimoxazole or doxycycline [22, 23]. Early clinical suspicion and continuation of appropriate treatment in this patient may have prevented her death.

## References

[1] Vandana KE, Mukhopadhyay C, Tellapragada C, Kamath A, Tipre M (2016). Seroprevalence of *Burkholderia pseudomallei* among adults in coastal areas in southwestern India. PLoS Negl Trop Dis.

[2] Peddayelachagiri BV, Paul S, Nagaraj S, Gogoi M, Sripathy MH (2016). Prevalence and identification of *Burkholderia pseudomallei* and near-neighbor species in the Malabar coastal region of India. PLoS Negl Trop Dis.

[3] John TJ (2004). Melioidosis, the mimicker of maladies. Indian J Med Res.

[4] Vishnu Prasad NR, Balasubramaniam G, Karthikeyan VS, Ramesh CK, Srinivasan K (2012). Melioidosis of chest wall masquerading as a tubercular cold abscess. J Surg Tech Case Rep.

[5] White NJ (2003). Melioidosis. The Lancet.

[6] Sugi Subramaniam RV, Karthikeyan VS, Sistla SC, Ali SM, Sistla S (2013). Intra-abdominal melioidosis masquerading as a tubercular abdomen: report of a rare case and literature review. Surgical Infections.

[7] Dhodapkar R, Sujatha S, Sivasangeetha K, Prasanth G, Parija SC (2008). *Burkholderia pseudomallei* infection in a patient with diabetes presenting with multiple splenic abscesses and abscess in the foot: a case report. Cases J.

[8] Cheng AC, Currie BJ (2005). Melioidosis: epidemiology, pathophysiology, and management. Clin Microbiol Rev.

[9] Limmathurotsakul D, Golding N, Dance DAB, Messina JP, Pigott DM (2016). Predicted global distribution of *Burkholderia pseudomallei* and burden of melioidosis. Nat Microbiol.

[10] Birnie E, Virk HS, Savelkoel J, Spijker R, Bertherat E (2019). Global burden of melioidosis in 2015: a systematic review and data synthesis. Lancet Infect Dis.

[11] Jesudason MV, Anbarasu A, John TJ (2003). Septicaemic melioidosis in a tertiary care hospital in south India. Indian J Med Res.

[12] Noyal MJC, Harish BN, Bhat V, Parija SC (2009). Neonatal melioidosis: a case report from India. Indian J Med Microbiol.

[13] Sharma G, Viswanathan S (2021). Melioidosis: a 5-year review from a single institution in Pondicherry. J Assoc Physicians India.

[14] Prakash A, Thavaselvam D, Kumar A, Kumar A, Arora S (2014). Isolation, identification and characterization of *Burkholderia pseudomallei* from soil of coastal region of India. Springerplus.

[15] Jose R, Valsan C, Sathiavathy KA (2019). Clinical and epidemiological profile of melioidosis in a tertiary care teaching hospital from South India. IJMMTD.

[16] Chang CY, Lau NLJ, Currie BJ, Podin Y (2020). Disseminated melioidosis in early pregnancy - an unproven cause of foetal loss. BMC Infect Dis.

[17] Rodríguez JY, Huertas MG, Rodríguez GJ, Vargas-Otalora S, Benıtez-Peñuela MA (2020). Case report: gestational elioidosis through perinatal transmission. Am J Trop Med Hyg.

[18] Porter MC, Pennell CE, Woods P, Dyer J, Merritt AJ (2018). Case report: chorioamnionitis and premature delivery due to *Burkholderia pseudomallei* infection in pregnancy. Am J Trop Med Hyg.

[19] Abbink FC, Orendi JM, de Beaufort AJ (2001). Mother-to-child transmission of *Burkholderia pseudomallei*. N Engl J Med.

[20] Currie BJ, Ward L, Cheng AC (2010). The epidemiology and clinical spectrum of melioidosis: 540 cases from the 20 year Darwin prospective study. PLoS Negl Trop Dis.

[21] Daim S, Barnad E, Johnny V, Suleiman M, Jikal M (2020). Neonatal melioidosis case reports-lessons learned. Clin Case Rep.

[22] Sullivan RP, Marshall CS, Anstey NM, Ward L, Currie BJ (2020). 2020 Review and revision of the 2015 Darwin melioidosis treatment guideline; paradigm drift not shift. PLoS Negl Trop Dis.

[23] Dance D (2014). Treatment and prophylaxis of melioidosis. Int J Antimicrob Agents.

